# Opium in Peritonitis

**Published:** 1879-02-20

**Authors:** R. M. Hand

**Affiliations:** Shubuta, Miss.


### *OPIUM IN PERITONITIS.*

BY R. M. HAND, M.D., OF SHUBUTA, MISS.

No one can now claim originality in the mere use of opium in peritonitis, for it has been employed to alleviate the pain of this disease from time immemorial, and latterly has been more or less depended on, in combination with other remedial agents, as a curative agent; but I wish to relate two cases, one of idiopathic and one of puerperal peritonitis, in which opium alone, in very large doses, was used with the most satisfactory results.

The first case I saw on the 25th of September, 1875; the patient was a large, robust man, about thirty years of age. I immediately commenced administering full doses of opium, two grains every two hours. This producing no sensible effect, I gradually increased the dose, watching closely, until nine grains were taken every two hours. The opium gave out during the treatment, and I substituted laudanum and morphia in equivalent doses. No less than this would produce the desired effect, that of relieving the pain and producing perfect quietude. This man in seven days took seven hundred and thirty-six grains of the drug, and made an excellent recovery. The second case, which I saw on the 23d of June, 1876, was a healthy multiparous female, about thirty-five years old, who was attacked, after a recent confinement, with pelvic peritonitis. I commenced, as before, with two grains of opium every two hours, and increased carefully until a dose of eight grains was reached, upon which she was kept for five days, at the end of which time it became necessary to diminish the dose, which was accordingly done in successively smaller doses as she improved, until the original quantity proved sufficient. She made an unusually early and satisfactory recovery.

It may be objected by some that, owing to the disordered state of the absorbents, caused by the inflammation, the medicine was not taken up. I would answer such by asking, how was it that the effects were proportionate to the size of the dose? If a dose of three grains produced no effect because it was not absorbed, would not one containing nine grains remained unabsorbed also? Some may be disposed to say that the opium used was not a good preparation; this objection can also be answered very conclusively, for be it remembered that in the first case the opium gave out, when morphine and laudanum were substituted. I was taught by a sad experience that the drug used in the second case was an active preparation. Having occasion to administer it to a stout, healthy woman, suffering pain from a recent uterine displacement, I left from the same bottle ten one-grain pills, with instruction to give one every hour until the pain was alleviated, but by all means not to arouse her to administer one. The caution was forgotten, and the two persevering relatives continued to shake and arouse her and force her to swallow, until she had taken seven pills, when I was sent for; but the most assiduous application of the usual remedies failed to avert the lethal effects of the opium.

I am satisfied that the above treatment is the best that has yet been advanced for the cure of peritonitis; yet, in recommending it, I would advise smaller doses to begin with and watchfulness in increasing, and to be on guard and ready to diminish as soon as it beeomes necessary. —*Med. and Surg. Reporter.*

